# Neural network-based prediction of atrial fibrillation at discharge following cardiac surgery

**DOI:** 10.3389/fcvm.2026.1886483

**Published:** 2026-08-05

**Authors:** Spela Leiler, Wolfgang Hitzl, Andre Bauer, Valentin Guenzler, Theodor Fischlein, Tomas Holubec, Jurij Matija Kalisnik

**Affiliations:** 1 Department of Cardiac Surgery, Klinikum Nuernberg, Paracelsus Medical University, Nuremberg, Germany; 2 Paracelsus Medical University, Nuremberg, Germany; 3 Department of Ophthalmology and Optometry, Paracelsus Medical University, Salzburg, Austria; 4 Research Program Experimental Ophthalmology and Glaucoma Research, Paracelsus Medical University, Salzburg, Austria; 5 College of Computing, Illinois Institute of Technology, Chicago, IL, United States; 6 Department of Internal Medicine II – Cardiology, Pneumology and Intensive Care, University Hospital Regensburg, Regensburg, Germany; 7 Faculty of Medicine, University of Ljubljana, Ljubljana, Slovenia; 8 Department of Cardiothoracic and Vascular Surgery, University of Graz, Innsbruck and Vienna Affiliated Tertiary Care Centre Klinikum KABEG, Klagenfurt, Austria

**Keywords:** atrial fibrillation, cardiac surgery, machine learning, neural networks, risk prediction

## Abstract

**Background:**

Postoperative atrial fibrillation is a common complication after cardiac surgery, associated with both short- and long-term adverse effects. Persistent forms of postoperative atrial fibrillation lasting until hospital discharge are less frequent but clinically significant. Despite its impact, reliable prediction remains challenging. This study aimed to develop and evaluate machine learning models that integrate electrocardiographic and clinical variables to predict atrial fibrillation at discharge.

**Methods:**

In this retrospective single-center study, 1,905 patients undergoing cardiac surgery were analyzed. Perioperative 12-lead electrocardiographic parameters and clinical variables were preselected using univariable logistic regression (*p* < 0.1). Various machine learning models, including neural networks, support vector machines, k-nearest neighbors' random forests, and Bayesian classifiers were trained, using 10-fold cross-validation and subsequently evaluated on an independent test set. A genetic algorithm was applied for variable selection, and a reject option was implemented to withhold uncertain predictions.

**Results:**

Of 1,905 patients 2.2% were discharged in atrial fibrillation. The final neural network model yielded a 69.9% coverage rate, with 99.2% of all predictions being correct (95% CI: 98.6–99.6). Key predictors included age, CHADS_2_-VASc score, left ventricular ejection fraction, EuroSCORE II, cardiopulmonary bypass time, prior cardiogenic shock, perioperative atrioventricular block, and mitral valve surgery.

**Conclusion:**

A machine learning model integrating perioperative electrocardiographic and clinical variables demonstrated robust performance in predicting the absence of atrial fibrillation at discharge, accurately identifying more than two thirds of patients. For the remaining patients, management continued to rely on clinical judgment. This approach offers a valuable tool for improved risk stratification and may facilitate the implementation of more targeted prophylactic strategies following cardiac surgery.

## 1 Introduction

Atrial fibrillation (AF) is a common postoperative complication following cardiac surgery, affecting 20%–60% of patients, and is typically converted to sinus rhythm before discharge through pharmacological therapy and/or electrical cardioversion (1, 2). However, a small subset of patients is discharged with persistent arrhythmia, despite appropriate treatment and no AF history. The incidence and management of AF at discharge remain not clearly defined, with limited available evidence (3, 4). The SEARCH-AF trial suggested that AF within 28 days after surgery has uncertain long-term significance and AF beyond this period may reflect persistent disease and a more complex underlying substrate (3, 5–7). Further evidence links treatment-resistant and long-lasting postoperative AF (>2 days) to increased heart failure hospitalization, but its association with higher mortality or thromboembolic risk remains unclear (8).

Persistent AF beyond discharge is an unpredictable event which complicates risk stratification (1). Conventional risk factors, including age and comorbidities, show limited predictive value (9). Advances in electrocardiographic analysis—particularly P-wave morphology and atrial conduction in combination with machine learning improving prediction of AF (10–15).

This study introduces artificial intelligence-based models integrating perioperative 12-lead ECGs and clinical variables to predict discharge AF. The aim was to differentiate among low-risk and high-risk patients to support personalized interventions, reduce complications, and optimize resource use.

## 2 Materials and methods

### 2.1 Study population

This retrospective observational study analyzed elective cardiac surgery cases at Klinikum Nürnberg (Germany) from January 2015 to January 2021, including aortic valve replacement (AVR), coronary artery bypass grafting (CABG), mitral valve replacement or reconstruction (MVR/MVr), tricuspid valve reconstruction (TVR), ascending aorta replacement, and their combinations. Subgroup analyses focused on 1) MVR/MVr with any other procedure and 2) AVR with any procedure excluding MVR/MVr. Inclusion criteria were age ≥18 years and preoperative sinus rhythm. Exclusion criteria included preexisting AF, missing ECG data, amiodarone use within the last month, advanced atrioventricular block (AVB) (IIb/III), pacemaker/defibrillator, emergent surgery, endocarditis, off-pump, or prior cardiac surgery.

### 2.2 Definitions

AF was defined as an irregular rhythm with absent *P*-waves lasting >30 s (16). Interatrial block (IAB) was identified by *P*-wave duration ≥120 ms in leads II, III, and aVF, and classified by morphology (17). Preexisting AF referred to any documented episode prior to surgery. Pulmonary hypertension was defined as a pulmonary artery systolic pressure >35 mmHg on echocardiography (18). Cardiogenic shock was diagnosed by evidence of end-organ hypoperfusion and/or hypotension (19). Cardiac surgery-associated acute kidney injury (CSA-AKI) was defined as a creatinine increase ≥0.3 mg/dL within 48 h or ≥1.5× baseline within 7 days postoperatively (20, 21).

### 2.3 Data collection

Medical records of 3,405 patients were reviewed, and perioperative ECGs were systematically analyzed for arrhythmia and conduction abnormalities. Standard 12-lead ECGs were obtained preoperatively, recorded daily during the early postoperative period (first 4 days), and repeated at hospital discharge. In addition, continuous cardiac rhythm monitoring was performed for at least the first 4 postoperative days and extended in the presence of arrhythmias.

### 2.4 Statistical analysis

Data underwent thorough editing to address incorrect and incomplete data. Continuous variables were assessed for normal, log-normal, and gamma distributions. Categorical variables were analyzed using Fisher's Exact and Pearson's chi-squared tests. Univariable logistic regression models provided odds ratios with 95% confidence intervals, scaled per 10-year age increase and per 10-minute CPB time for interpretability. Variable selection was guided by clinical expertise (1, 2, 13, 14, 21) to achieve a set of candidate variables, and to be optimized by a genetic variable selection algorithm. Only complete cases were included in the final modelling dataset. Shapley values were used to illustrate feature importance and each risk factor's contribution to AF prediction at discharge. Machine learning models including multilayer perceptron networks, support vector machines, k-nearest neighbors, random forests, and Bayesian classifiers, were trained using prior probabilities. Model development involved preprocessing, data partitioning (training, validation, test), 10-fold cross-validation, and testing in an independent test sample. A reject option based on two posterior probability thresholds was used to withhold uncertain predictions. Model performance was evaluated using positive and negative predictive values (PPV, NPV), coverage rate (proportion of all patients for which the model provides a prediction, rather than refusing to answer) and proportion of total correctly predicted cases, across training, test and overall samples. Statistical significance was defined as *p* < 0.05 (two-sided). This study was conducted and reported in accordance with TRIPOD guidelines for prediction model studies (22).

### 2.5 Model reproducibility

Analyses were performed using STATISTICA 13 and MATHEMATICA 13.0 (23, 24). Models were trained using 10-fold cross-validation and evaluated on an independent test set, weights were initialized randomly with fixed random seeds. Hyperparameters were optimized via a stochastic search process which combines a random search across the hyperparameter space with an underlying Bayesian optimization strategy using Gaussian process priors. To counter the inherent risks of overfitting associated with a low-prevalence outcome (2.2% event rate), weight regularization was strictly enforced. An L2 regularization (weight decay) penalty was integrated into the loss function. During the 10-fold cross-validation phase, model training was continuously monitored against the validation fold. Training was automatically terminated via an early stopping protocol when the validation loss ceased to decrease or began to diverge from the training loss. This prevented the network from over-optimizing the training data and preserved its predictive power for the independent test set. The network architecture consists of 11 consecutive layers, incorporating a concatenation layer, multiple linear layers, hyperbolic tangent and logistic sigmoid activations, and a final Softmax normalization layer to convert raw outputs into posterior probabilities. Variable selection was performed using a genetic algorithm, and prior probabilities were used to incorporate existing knowledge about class frequencies.

### 2.6 Ethical statement

The study complied with the Declaration of Helsinki and the Oviedo Convention. It was approved by the institutional review board of Paracelsus Medical University (IRB-2020-028) on January 5, 2021. Due to its retrospective design and full anonymization, informed consent was waived.

## 3 Results

**Figure 1:**
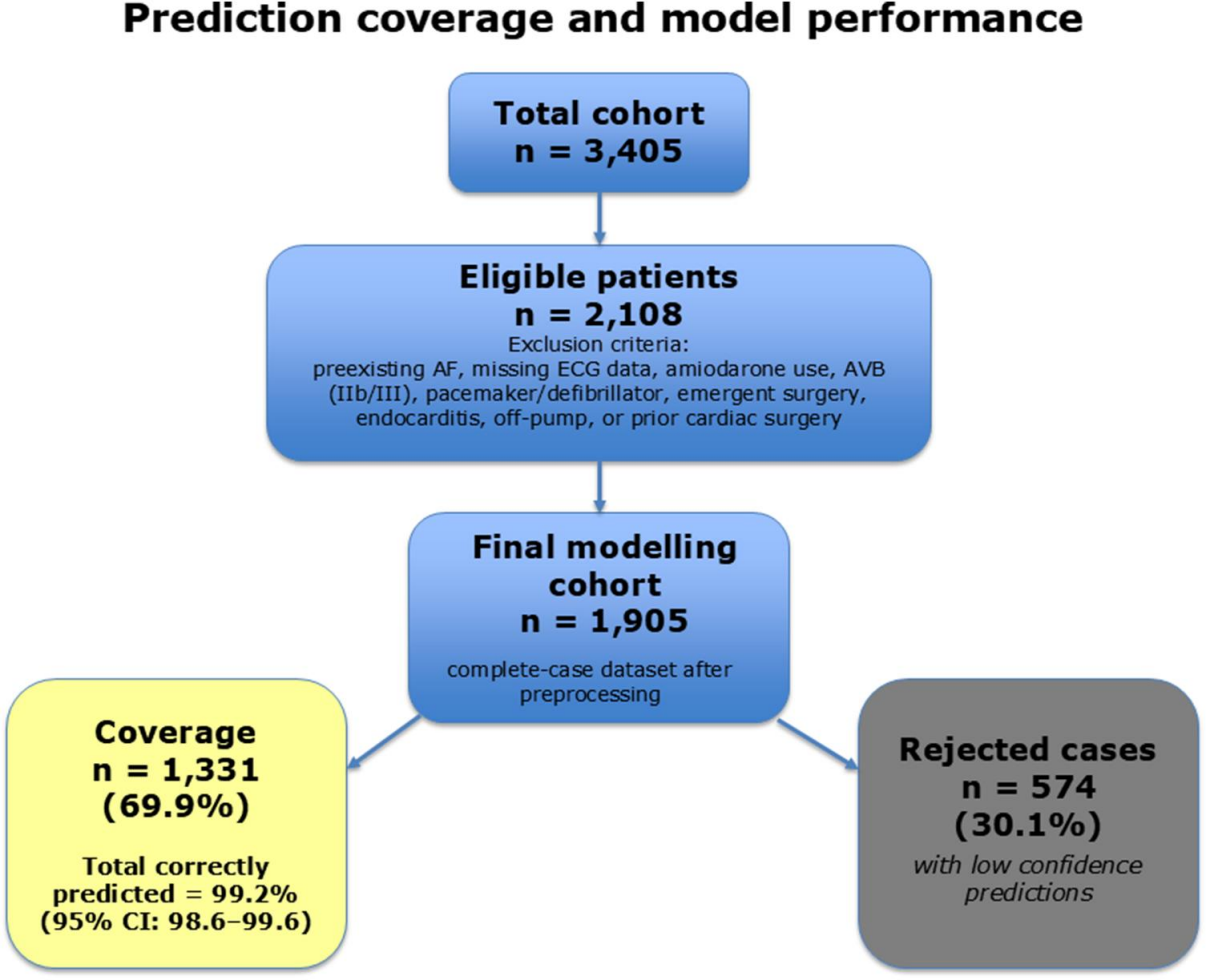
Flow diagram of cohort selection and model prediction coverage.

**Figure 2:**
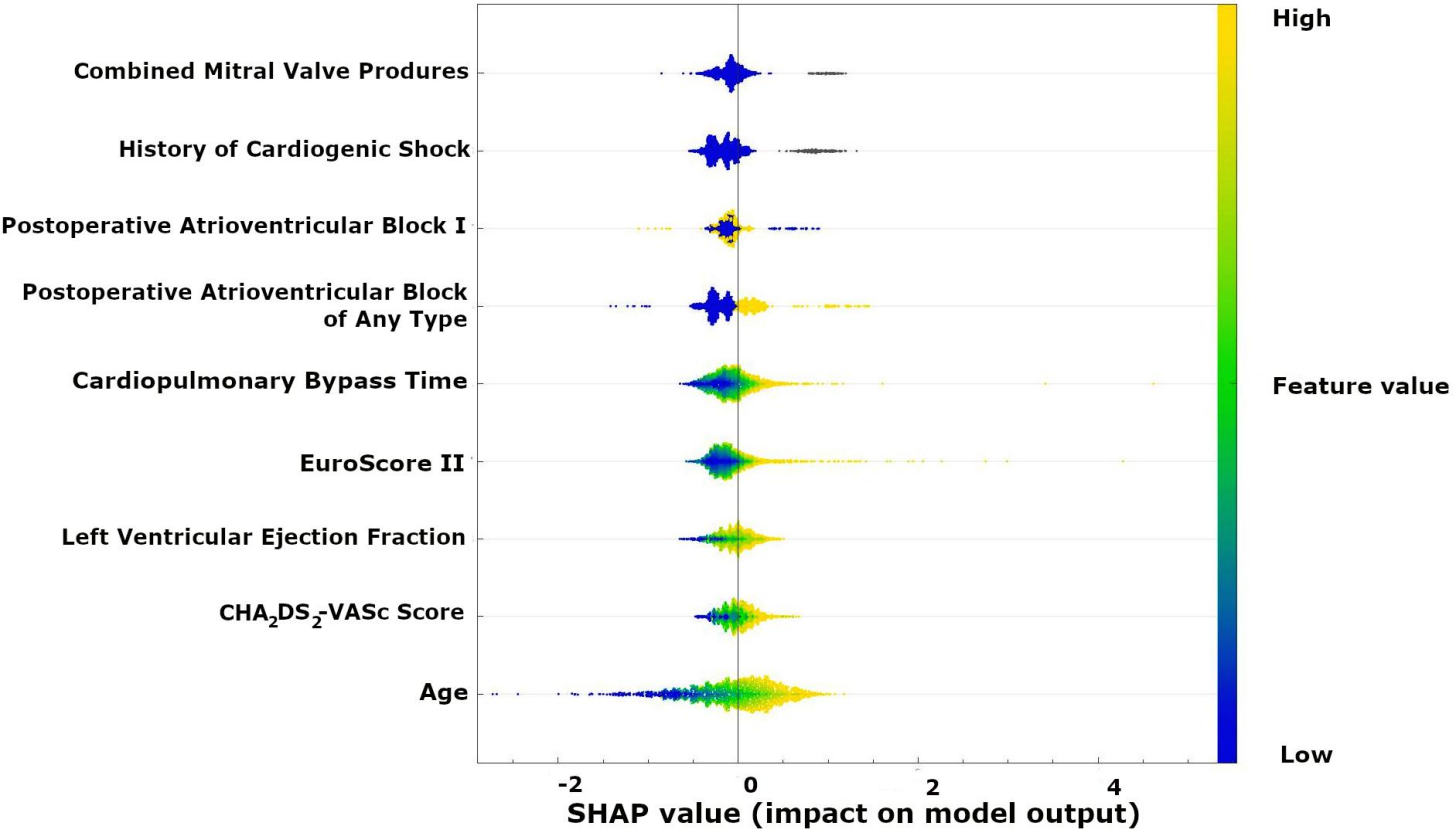
Shapley graph for predictors for atrial fibrillation at discharge. Shapley values were used to illustrate feature importance and each risk factor's contribution to AF prediction at discharge. The *x*-axis shows the marginal effect on predicted probability, with color indicating variable magnitude (yellow=high, blue=low).

At least one episode of AF was documented in 765 patients (36.3%) during hospitalization, and forty-two patients (2.2%) were discharged in AF. Among 2,108 available for the analysis, 1,905 patients were included in the final modelling (Figure 1). An overview of patient characteristics with and without AF at discharge and corresponding univariable odds ratios are presented in Table 1. Performances in the training, test and overall sample before applying the reject option are presented in Table 2. The neural network model yielded a 69.9% coverage rate, with 99.2% of all predictions being correct (95% CI: 98.6–99.6) (Table 3). The reject option resulted in withholding predictions in 30.1% of cases, corresponding to instances with lower model confidence. The Shapley values of the final model and key predictors are presented in the Figure 2.

**Table 1:** Overview of baseline characteristics, clinical parameters and their impact on atrial fibrillation at discharge in terms of univariable odds ratios.

Baseline characteristics and univariable predictors of atrial fibrillation at discharge	No AF at discharge group, *n* = 2,060 Mean (SD)/n (%)	AF at discharge group, *n* = 48 Mean (SD)/n (%)	*p*-value	OR [95% CI]
Age	66.7 (9.7)	72 (7.1)	<0.001	1.70 [1.30–11.20]
Gender (Woman)	574 (27.9%)	20 (41.7%)	0.033	1.80 [1.02–3.28]
CHA_2_DS_2_-VASc Score	3.0 (1.5)	3.5 (1.4)	0.023	1.24 [1.03–1.50]
LV-EF (%)	56.1 (10.6)	56.4 (10.1)	0.85	1.00 [0.97–1.03]
EuroSCORE II	2.5 (2.1)	4.4 (4.3)	<0.001	1.18 [1.09–1.26]
History of cardiogenic shock	162 (7.9%)	12 (25.0%)	<0.001	4.21 [2.12–8.39]
Pulmonary hypertension	84 (4.1%)	8 (16.7%)	<0.001	4.96 [2.23–11.05]
NYHA III/IV	995 (48.3%)	27 (56.2%)	0.16	1.58 [0.84–2.99]
COPD (GOLD 3/4)	273 (13.3%)	8 (16.7%)	0.42	1.38 [0.63–3.00]
Preoperative Creatinine >1.4 mg/dL	135 (6.6%)	4 (8.3%)	0.57	1.35 [0.48–3.85]
Cardiopulmonary bypass time (Min.)	93 (43.7)	117.1 (54.7)	<0.001	1.05 [1.01–1.09]
β-blockers	986 (47.9%)	27 (56.2%)	0.27	1.38 [0.77–2.46]
MRA Antagonists	122 (5.9%)	3 (6.2%)	0.93	1.05 [0.32–3.44]
Preoperative IAB	728 (35.3%)	27 (56.2%)	0.003	2.33 [1.31–4.15]
Preoperative AVB I and IIa	248 (12.0%)	9 (18.8%)	0.17	1.67 [0.80–3.50]
Preoperative LBBB	75 (3.6%)	3 (6.2%)	0.35	1.75 [0.53–5.77]
Preoperative RBBB	105 (5.1%)	4 (8.3%)	0.97	1.68 [0.59–4.77]
Postoperative IAB	566 (27.5%)	20 (41.7%)	0.033	1.87 [1.04–3.34]
Postoperative AVB	661 (32.1%)	25 (52.1%)	0.004	2.28 [1.29–4.05]
Postoperative AVB I	612 (29.7%)	16 (33.3%)	0.61	1.17 [0.64–2.15]
Postoperative AVB IIa	15 (0.7%)	2 (4.2%)	0.009	5.89 [1.31–26.52]
Postoperative AVB IIb	9 (0.4%)	0 (0.0%)	0.65	-
Postoperative AVB III	70 (3.4%)	9 (18.8%)	<0.001	6.51 [3.04–13.97]
Intervention for bleeding	67 (3.3%)	5 (10.4%)	0.044	2.83 [0.98–8,16]
CSA-AKI	269 (13.1%)	14 (29.2%)	0.001	2.72 [1.44–5.14]
Isolated AVR	512 (24.9%)	9 (18.8%)	0.32	0.69 [0.33–1.43]
Isolated MVR/r	74 (3.6%)	8 (16.7%)	<0.001	5.33 [2.41–11.80]
Isolated TVr	3 (0.1%)	0 (0%)	1.0	-
Isolated CABG	839 (40.7%)	6 (12.5%)	<0.001	0.21 [0.09–0.49]
Isolated Aortic Surgery	37 (1.8%)	0 (0.0%)	0.37	0.97 [0.97–0.98]
Combined AVR	359 (17.4%)	12 (25.0%)	0.18	1.57 [0.81–3.04]
Combined MVR/r	92 (4.5%)	7 (14.6%)	0.001	3.62 [1.58–8.30]
Bicaval cannulation	169 (8.2%)	15 (31.2%)	<0.001	5.05 [3.69–9.49]

**Table 2:** Overview of the results in terms of total accuracy of five machine learning algorithms.^a^

Atrial fibrillation at discharge	Performance of machine learning models for predicting atrial fibrillation at discharge	Support vector machine	K-nearest neighbours model (KNN)	Random forest model	Bayes classifier	Neural network
*n* = 1,333	training sample	98.0%	98.2%	98.0%	97.8%	97.4%
*n* = 572	test sample	97.2%	97.1%	97.2%	97.1%	98.6%
*n* = 1,905	overall	97.8%	97.8%	97.8%	97.1%	97.8%

a Results are obtained before application of reject option and are 10-fold cross-validated.

**Table 3:** Overview of model performances of the best neural network model to predict atrial fibrillation at discharge following cardiac surgery.^a^

Performance of the best neural network model for predicting atrial fibrillation at discharge	Negative predictive value PV	Positive predictive value	Coverage^b^	Total correctly predicted^c^
Results in the training sample (10-fold cross-validated)	(916/923) 99.2%	–	(923/1,333) 69.2%	(916/923) 99.2%
Results in the independent test sample	(405/408) 99.3%	–	(08/572) 71.3%	(405/408) 99.3%
Results in the overall sample	(1,321/1,331) 99.2%	–	(1,331/1,905) 69.9%	(1,321/1,331) 99.2%

a Results are obtained after applying the reject option and are reported in the training, independent test and overall samples to demonstrate the model's ability to generalize to new, previously unseen patients.

b Proportion of all patients for which the model provides a prediction, rather than refusing to answer.

c The percentage of correct outcomes out of all cases where the model actually made a prediction.

## 4 Discussion

### 4.1 Principal findings

To our knowledge, this is among the first studies presenting a personalized prediction model for AF at discharge demonstrating such high predictive performance (25) with NPV of 99.2% (95% CI: 98.6–99.6) in 69.9% cases including the following predictors: advancing age, CHA_2_DS_2_-VASc score, EuroSCORE II, left ventricular ejection fraction, prior cardiogenic shock, perioperative atrioventricular block, mitral valve surgery and cardiopulmonary bypass time.

### 4.2 Comparison with existing literature

Advanced age is a principal determinant of AF as age-related atrial remodeling—characterized by fibrosis, conduction delay, arterial stiffening, and diastolic dysfunction—creates a pro-arrhythmic substrate independent of conventional risk factors (1, 2, 13, 15). In cardiac surgery, perioperative inflammation and heightened sympathetic activity may exacerbate this substrate (26, 27). CHA_2_DS_2_-VASc in cardiac surgery reflects preexisting comorbidities that may predispose patients to postoperative AF, suggesting its potential role in identifying those at higher risk for AF (28). Similarly, in cardiology setting, a CHA_2_DS_2_-VASc score ≥2 predicts AF recurrence after pulmonary vein isolation (PVI) and correlates with new-onset AF (29, 30). Additionally, reduced left ventricular ejection fraction and prior cardiogenic shock further reflect structural remodeling associated with increased AF risk (31, 32).

Postoperative AVB especially first-degree AVB, is a significant predictor of AF with studies showing a ∼2-fold increased AF risk (33). PR-Interval has previously been used as a risk estimator in Framingham AF risk score (34). In the postoperative setting, atrioventricular conduction disturbances may arise because of surgical trauma, inflammation, edema, and ischemia affecting the cardiac conduction system. Underlying factors include advanced age, number of bypassed vessels, valve surgery, prolonged aortic cross-clamp time, pre-existing conduction abnormalities, and the use of antiarrhythmic therapy (e.g., amiodarone, sotalol) (35). These influences can impair atrioventricular nodal and atrial conduction, often reflected by PR prolongation. Although the relationship between atrioventricular conduction delay and AF remains controversial (15, 33), both conditions may reflect overlapping pathophysiological mechanisms, including atrial structural remodeling and conduction heterogeneity (36). In this context, PR prolongation may be better interpreted as a marker of underlying atrial cardiomyopathy rather than a direct causal factor in AF development.

Mitral valve surgery is strongly associated with postoperative AF, likely due to atrial structural remodeling from valvular pathology (e.g., dilatation, fibrosis) and the invasive nature of the procedure (1, 2, 37, 38). The role of cardiopulmonary bypass (CPB) duration in postoperative AF remains controversial. CPB induces a systemic inflammatory response via blood–artificial surface interaction, surgical trauma, and ischemia–reperfusion injury (7, 32). Comparable AF incidence in off-pump surgery suggests that surgical manipulation alone may similarly trigger inflammatory activation and cytokine release (39).

### 4.3 Clinical implications

Traditional clinical risk scores, though simple and widely applicable, offer only moderate predictive accuracy. The CHA_2_DS_2_-VASc demonstrated stronger diagnostic performance (AUC 0.77, 95% CI 0.75–0.79), followed by the POAF score with moderate discrimination (AUC 0.71, 95% CI 0.69–0.73) (2, 40). In another study Chen et al. conducted a meta-analysis including 18,086 patients and found also found that CHA_2_DS_2_-VASc score is an independent predictor of POAF (OR: 1.46; 95% CI: 1.25 1.72) (41). In contrast to conventional prediction models, our approach focuses on making highly reliable, individualized predictions by incorporating a reject option, meaning the model withholds a prediction when uncertainty is high. As a result, measures such as negative predictive value and coverage were considered more informative than overall performance metrics like the area under the curve. Artificial intelligence–based models, which integrate complex clinical data using advanced computational methods, can achieve improved predictive performance and may represent an important step forward in risk stratification for atrial fibrillation (12, 25, 42). A recent artificial intelligence–based prediction model achieved excellent discrimination (AUC 0.931), substantially outperforming conventional clinical risk scores (4).

Current guidelines (16) provide limited specificity regarding AF management after cardiac surgery, largely recommending amiodarone without stratification based on individual risk profiles. Preventive strategies should instead be directed toward patients with elevated risk, including those with advanced age, structural heart disease (particularly atrial enlargement and mitral valve pathology (1, 2, 31), pre-existing conduction abnormalities (13–15, 43), prolonged cardiopulmonary bypass or cross-clamp times, and signs of heart failure or systemic inflammation (2, 7, 44, 45). The personalized prediction model presented in this study enables identification of a substantial low-risk subgroup with high certainty, supporting its use as a screening tool to guide de-escalation of therapy. In these patients, standard postoperative care may be sufficient, thereby reducing unnecessary exposure to antiarrhythmic and anticoagulant therapies and their associated adverse effects, including amiodarone-related pulmonary or hepatic toxicity and bleeding complications (46).

Conversely, in patients in whom low risk cannot be reliably established, targeted prophylactic strategies should be considered. These include early initiation or reintroduction of β-blockers, selective use of perioperative amiodarone, and anti-inflammatory approaches such as colchicine (47), alongside with optimization of electrolyte balance and hemodynamic status. In addition, procedural interventions such as posterior left pericardiotomy or targeted pericardial drainage, which facilitate pericardial fluid clearance and have been associated with reduced AF incidence (16, 48), may be selectively applied in higher-risk cohorts. By capturing complex, nonlinear interactions among clinical variables, the model demonstrates improved predictive performance compared with conventional risk scores and supports a risk-adapted, individualized approach to postoperative AF prevention.

### 4.4 Strengths and limitations

By implementing the dual threshold reject option, the model withheld predictions for 30.1% of highly uncertain cases. For the remaining 69.9% of accepted cases, it achieved a NPV of 99.2% (95% CI: 98.6–99.6). This mathematical shift demonstrates that the reject option successfully filters out clinical “noise” and high-uncertainty cases, resulting in a clinical tool that can rule out discharge AF with near-perfect certainty for nearly 70% of the surgical population.

This single-centre, retrospective study in an elective surgical population has inherent limitations due to the retrospective character of the study without external validation. However, this approach was necessary to accumulate a large enough cohort (*n* = 3,405) to model a rare 2.2% outcome. To mitigate retrospective bias and overfitting, we utilized strict data segregation and evaluated the model on an independent test set. While these results confirm the model's high technical accuracy, prospective multi-centre validation is required before clinical implementation. Future studies utilizing external validation on independent geographic or institutional cohorts are required to confirm the model's performance across diverse clinical settings before routine implementation.

Valuable clinical data were not available for the analysis, such as of left atrial size (2), perioperative electrolyte levels (1), haemoglobin and ferritin levels (49), additional ECG parameters including advanced heart rate dynamics analyses (15), standardized definitions of heart failure, and documentation of postoperative complications; such as retained blood syndrome, pericardial effusion, and chest tube placement (48, 50, 51). Information regarding the duration and dosage of preoperative medical therapy, as well as perioperative medication adjustments and postoperative pharmacological management as well as laboratory values, was not consistently available in our retrospective dataset. These factors may influence the development of postoperative atrial fibrillation and should be incorporated into future prospective studies to further improve the predictive performance and clinical applicability of machine learning models.

Further analyses are necessary to differentiate true risk factors from confounders such as cardiopulmonary bypass duration and surgical complexity. The reject option, while enhancing model performance, resulted in 30.1% of patients without a prediction, still requiring clinician judgment. Furthermore, this study assumes that AF at discharge reflects a more advanced arrhythmic substrate and increased risk of chronic AF. Electrophysiological studies in cardiac surgery patients, continuous rhythm monitoring throughout hospitalization, and comprehensive long-term follow-up after discharge are essential to confirm these assumptions and to better characterize the transition from postoperative AF to persistent arrhythmia. Such prospective investigations would also facilitate refinement of the present prediction model and improve its clinical applicability for individualized patient management.

## 5 Conclusion

This personalized model predicts the absence of AF at discharge with high accuracy (NPV 99.2%), enabling safe identification of low-risk patients. It can help tailoring prophylactic strategies, reducing unnecessary therapies, and optimizing postoperative resource use. Further refinement using multicenter data and exploration of predictor-outcome relationships is warranted. Expanding the model to predict AF recurrence and long-term outcomes, along with early integration of wearable monitors and digital ECGs, may further improve detection and guide resource allocation.

## Data Availability

The raw data supporting the conclusions of this article will be made available by the authors, without undue reservation.
